# Aligning research agendas and evidence on violence against women and children in low- and middle-income countries: Lessons from a pilot mapping approach

**DOI:** 10.1186/s12961-026-01518-w

**Published:** 2026-08-13

**Authors:** Amy R. Riley-Powell, Yulidsa Angie Bedoya Zuniga, Shanice Eksteen, Mark Tomlinson, Elizabeth Dartnall

**Affiliations:** 1 Institute of Development Studies, University of Sussex, Brighton, United Kingdom; 2 Sexual Violence Research Initiative, Pretoria, Gauteng South Africa; 3 Utrecht University, Utrecht, The Netherlands; 4 Department of Global Health, Institute for Life Course Health Research, Stellenbosch University, Cape Town, South Africa; 5 School of Nursing and Midwifery, Queens University, Belfast, United Kingdom

**Keywords:** LMICs, Monitoring and evaluation, Research, Research agenda, Research priorities, VAW, VAC

## Abstract

**Background:**

One in three women globally experience physical or sexual violence, driving the urgent need for research to inform prevention and response efforts. The Global Shared Research Agenda (GSRA), developed by the Sexual Violence Research Initiative (SVRI) and the Equality Institute (EQI), sets out 41 priority research questions across four domains for addressing violence against women (VAW) and violence against children (VAC) in low- and middle-income countries (LMICs): (1) understanding violence against women and girls in its multiple forms, (2) intervention research, (3) improving existing interventions and (4) methodological and measurement gaps. Yet, there are few practical tools for assessing whether research activity is meaningfully aligned with these priorities. This study pilots a desk-based mapping approach to examine how research presented at the SVRI Forum aligns with the GSRA.

**Methods:**

To address this gap, we piloted a desk-based mapping approach using all abstracts accepted at the 2019 and 2022 SVRI Forums; large, peer-reviewed, abstract-driven conferences focused on VAW and VAC. While not exhaustive of all research underway, SVRI Forum abstracts provide a structured, time-bound snapshot of current research trends, notably in LMICs. Grounded in an interpretivist approach, we used a qualitative content-mapping method informed by directed content analysis. Titles and abstracts were systematically matched to the 41 GSRA priority research questions to identify patterns of alignment, gaps and emerging themes.

**Results:**

Most abstracts aligned with at least one GSRA priority question, with the greatest concentration in Domain 1 (understanding C in their multiple forms) and Domain 3 (improving existing interventions). Fewer abstracts addressed priority research questions related to lesbian, gay, bisexual, trans, queer, intersex, asexual, and more (LGBTQIA+) populations, justice sector responses and long-term or intersectional measurement. Unmatched abstracts highlighted emerging themes, which include decolonization, practitioner well-being and political and funding dynamics, which fall outside the current GSRA scope and signal areas where priorities may be evolving. The process also underscored other, broader challenges with research priority question formats and the level of specificity required for research agendas.

**Conclusions:**

Findings suggest that the research presented at the SVRI Forums is broadly aligned with GSRA research priorities, while also highlighting areas where the agenda may require periodic updates to reflect emerging themes and methodological needs. The mapping approach offers an accessible tool for tracking the influence of research agendas such as the GSRA. Embedding similar monitoring mechanisms within research convenings may strengthen feedback loops between agenda-setting, funding decisions and practice, helping ensure that global research priorities remain responsive and relevant to stakeholders.

## Introduction

One in three women experience physical or sexual violence in their lifetime – a profound violation of human rights with devastating impacts on survivors, families and communities [49]. This violence restricts women’s freedoms and limits national development by reducing women’s participation in education and the workforce, increasing health and social service costs and perpetuating cycles of poverty and inequality [5, 49]. Violence against children (VAC) is similarly widespread, with significant consequences for health, well-being and intergenerational cycles of harm and often intersects with violence against women within families and communities [18, 33, 50]. The importance of addressing violence against women (VAW) and VAC is represented on the global agenda through inclusion in the Sustainable Development Goals (Goal 5), international human rights frameworks, such as the Convention on the Elimination of All Forms of Discrimination against Women [9], along with United Nations (UN) initiatives, and regional and grassroots advocacy campaigns [14, 45]. With increased commitment to preventing and responding to VAW and VAC, building consensus on shared priorities has never been more important.

Research agendas are powerful advocacy tools which can be used by stakeholders, policy-makers, funders and organizations to highlight focus areas and galvanize commitment towards shared goals [8, 15, 17, 23]. Unlike general advocacy campaigns or policy statements, research agendas are structured to define research priorities, coordinate stakeholders and guide investment in evidence generation. One example is the Global Shared Research Agenda (GSRA), developed to address VAW and VAC in low- and middle-income countries (LMICs) [36]. The GSRA was the first global shared research agenda to address VAW and VAC, adopting a forward-looking approach that shifted the focus from response to prevention. The GSRA outlines four key areas where evidence is lacking, called domains, each of which contain a set of research questions that collectively represent the research priorities. The four domains cover (1) understanding violence against women and girls in its multiple forms, (2) intervention research, (3) improving existing interventions and (4) methodological and measurement gaps. These domains collectively structure the 41 priority research questions that form the GSRA, which are detailed in Table 1.

**Table 1:** The GSRA domains and questions

GSRA domain	Research questions
Domain 1: Understanding violence against women and girls in its multiple forms, including prevalence of different types of VAW, risk and protective factors for VAW experience and perpetration and the causes and consequences of VAW, including health and psychosocial consequences	Question (Q) 3: How are new feminist social movements (for example, Me Too, Ni una menos) and meninist social movements [Men’s Rights Activists (MRAs), incels, etc.] positively or negatively influencing individual, social and policy perspectives related to the experience and perpetration of violence? Question 5: What are the impacts (including disability-related impacts) of under-researched forms of intimate partner violence (IPV) on women and girls, including emotional and economic IPV, revenge porn and “honour”-based violence? Question 7: What are the cultural, psychological and economic impacts of colonization on Indigenous men and women, and how do these impacts influence their behaviours and experiences in respect to VAW? Question 16: How do conflict and fragility exacerbate the multiple forms of violence experienced by women and girls? Question 30: How do different forms of violence cluster in women and girls with greater vulnerability, and what are characteristics to detect those vulnerable women and girls? Question 31: What are the causes and drivers of violence against LGBTQI+ women? Question 35: What is the interaction of climate change impacts with the perpetration or experience of VAW? Question 37: What is the prevalence of different forms of online and technology-facilitated VAW, and what are the risk and protective factors for experience and perpetration of these types of violence? Question 39: What steps can be taken to avoid or mitigate resistance to and backlash against women’s rights organizations without compromising the focus and aims of these organizations? Question 41: How do social networks act as a protective factor for violence against women and girls?
Domain 2: Intervention research, including research on violence prevention and response interventions, and various types of evaluations of interventions, including process, formative and impact evaluations	Question 1: What types of interventions can effectively prevent multiple forms of violence, and why? Question 2: What types of interventions are most effective for preventing intimate partner violence (including “honour”-based violence) against women facing multiple and intersecting forms of discrimination (including age, poverty, disability, ethnicity, race and sexuality)? Question 4: What interventions work to prevent sexual harassment in institutional settings (in-person or online), including in the workplace and educational settings, and why? Question 6: What is the level of intensity needed for social norms change interventions to have sustained impact at the community level, including effectively challenging norms that focus on victim behaviour rather than on the perpetration of/choice to use violence? Question 8: What role can formal and informal justice sector reforms, including restorative justice, play in ensuring justice for survivors of violence? Question 9: What interventions or elements of interventions are most effective at preventing violence against adolescent girls, and why? Question 11: Which interventions are most effective at addressing shared risk factors for VAW and VAC in the family environment, leading to a reduction in both types of violence? Question 26: In what ways can innovative technologies and interventions be used to detect and prevent online sexual harassment and online intimate partner violence? Question 29: What are the factors underlying successful intervention and prevention programmes aimed at men, including Indigenous men and other under-researched populations? Question 36: What types of interventions are effective in preventing IPV and other forms of violence against LGBTQIA+ people?
Domain 3: Improving existing interventions, including scale-up research, costing research, intervention science, process research and other forms of research that generate innovative solutions to improve existing interventions, making them more deliverable, affordable or sustainable including research aimed at understanding the impact of policies and laws on VAW	Question 17: What alternative modalities (beside in-person programming) are effective in VAW prevention at scale? Question 18: How can large-scale sector programmes be adapted to optimize their impact on violence prevention and response, particularly education, health, economic development, infrastructure and social protection programmes? Question 19: How can social movements and feminist activism contribute to preventing and responding to VAW at scale? Question 20: What are some best practices for ensuring agility and adaptability of VAW interventions, especially those working with marginalized women and girls or operating in complex contexts? Question 24: Do higher costs in resource-intensive violence prevention interventions represent good value for money when taking into account effectiveness in reduction of VAW? Question 25: How can promising VAW prevention and response interventions from non-emergency settings be adapted to have effect in conflict and humanitarian contexts (for example, reduced dosage or brevity, different delivery mechanisms)? Question 27: How can we use tech platforms effectively, safely and cost-efficiently for violence prevention? Question 28: In what ways can justice institutions be held to account and capacitated to be survivor-centred and hold perpetrators accountable, especially in conflict and post-conflict settings? Question 33: What types of interventions are most effective in facilitating gender-transformative change in men and women at scale? Question 38: How can police response more adequately address the needs of LGBTQIA+ people reporting IPV, non-partner sexual violence and sexual harassment? Question 40: What kinds of faith-based or community-led VAW prevention interventions can be adapted to different faiths, communities and regions effectively?
Domain 4: Methodological and measurement gaps, including new and innovative ways to measure VAW, hierarchies of knowledge, practice-based learning, sticky ethical issues and monitoring and evaluation of interventions	Question 10: What are the most effective tools to measure harmful traditional practices against women and girls [including female genital mutilation/cutting (FGM/C)], early and forced marriage, crimes committed in the name of honour, dowry-related violence and son preference)? Question 12: What methods can be used to measure the intersection and pathways between different types of violence, including polyvictimization and intersections between VAW and violence against children (VAC)? Question 13: How to conduct effective, ethical and inclusive research on VAW using online/virtual/remote methods (including social media), and how should these be adapted to reach marginalized populations? Question 14: What research methodologies are most appropriate to measure social norm change in violence prevention interventions? Question 15: In IPV prevention interventions inclusive of women and girls with disabilities, should outcome measures be universal, or should some be disability-specific? Question 21: What methodologies can be used to measure and attribute the impact of multi-component interventions on VAW prevention, reduction or cessation? Question 22: What are examples of good practice in addressing recognized ethical challenges of undertaking VAW research in resource-poor settings and/or with marginalized communities? Question 23: How do we ensure our research impacts policy and programmes, and how do we measure that impact? Question 32: What are the best methodologies to measure the long-term impacts of violence prevention interventions, including reduction in VAW and other intended and unintended outcomes? Question 34: Which analytical approaches (both quantitative and qualitative) are most appropriate for advancing an intersectional approach to research on VAW?

These research priorities are designed to support researchers, funders and policy-makers plan and allocate resources, inform SVRI grant-making, highlight important research topics to stakeholders and provide a structure for tracking research activity in the field [10, 36]. For the purposes of this study, we used the research questions as the basis for mapping and analysis. The domains and associated priority questions are presented in Table 1.

### Developing the global shared research agenda

In 2021, the Sexual Violence Research Initiative (SVRI), the world’s largest research network focused on VAW and VAC, partnered with the Equality Institute (EQI), a feminist agency dedicated to preventing VAW and VAC through research, communications and leadership, to develop the GSRA [12]. The GSRA’s development marked a milestone in progressive and participatory research methodology, reflecting a broader trend towards co-produced research agendas [38, 41, 42, 46]. Traditionally, research priority-setting has relied heavily on experts and centralized decision-making, often with limited involvement from affected communities and underrepresented groups [29, 43, 47]. In contrast, the GSRA intentionally centres voices traditionally marginalized in policy and research, including those with lived experience and communities from the Global South, broadening participation and challenging power imbalances in research agenda-setting [10, 47].

To develop the GSRA, an innovative adaptation of the Child Health and Nutrition Research Initiative (CHNRI) method was employed, leveraging crowdsourcing to engage a diverse pool of stakeholders including donors, academics, practitioners and individuals with lived experience [10, 29, 30]. The process involved six steps, guided by stewardship, advisory and global expert groups. Potential research domains were identified through a literature review, followed by the collection of suggested research questions via surveys and webinars. Global expert groups then validated and refined the domains and research questions, culminating in a final agenda comprising four domains, each containing 10–11 priority research questions [36]. Through this novel adaptation of the CHNRI method, the GSRA increased participation and further democratized the agenda-setting process, highlighting the potential to influence how global research priorities are established [10]. However, questions remain about their long-term impact on stakeholder engagement, research uptake and meaningful change in practice [47, 51].

The GSRA is one of several regional and thematic research agendas developed by the SVRI and partners, which build evidence to support progress towards eliminating all forms of VAW and VAC [28, 37–40, 47].

### How can we evaluate the influence of the GSRA?

To build on the shift towards more representative and inclusive research agendas, we need to consider how we track their influence. Tracking the impact of research agendas is limited, as noted by McGregor et al., who state that “in the absence of published information on implementation or evaluation, it is not possible to assess what the impact and effectiveness of health research priority-setting may have been” [25]. To fully leverage the significant time and financial investment in developing research agendas, we must address the entire process – from setting priorities (front end) to implementation and evaluation (back end). Key processes such as coordination, funding and stakeholder engagement are essential at multiple stages and play a crucial role in ensuring the agenda’s effectiveness and sustainability. Monitoring influence can be achieved through various methodologies, including evaluations. Evaluations can be used to assess the uptake, outcomes and broader impact of research agendas, while facilitating iterative feedback mechanisms that ensure responsiveness to real-world needs, promote knowledge exchange and strengthen the link between policy formulation and practical implementation [43].

Evaluation processes often rely on elaborate data collection methods, extensive reporting structures and high-level expertise, all of which can be burdensome for research teams and funders. There are some promising methods that offer alternatives to traditional evaluations. The Theory of Change (ToC) helps map how research is expected to create impact, making it easier to track outcomes over time [7, 48]. Real-time dashboards and feedback loops support continuous, low-burden monitoring, which enables more responsive and efficient evaluations [27]. Participatory processes also bring in relevant stakeholders to ensure evaluations are grounded in local context and reduce complexity [6, 19, 34].

A central principle of participatory evaluation is the prioritization of lived experience in achieving meaningful stakeholder alignment. Centring these perspectives enhances the relevance and practical utility of findings, complementing governance-driven approaches [1]. Such engagement supports research agendas that are responsive to real-world needs, without overburdening actors and stakeholders.

Despite these developments, there is limited literature examining the potential for participatory or adaptive evaluations within the context of research agendas [21].

The mapping method presented in this study offers a feasible alternative for monitoring alignment between the GSRA and research happening on the ground, creating a feedback loop to inform updates and ensuring the GSRA remains relevant. In this paper, we present a novel mapping method to monitor the impact of the GSRA by comparing it with VAW and VAC research being conducted globally. The aims of this paper are to:

1. Examine trends in VAW and VAC research over 3 years and explore the alignment and convergence between the GSRA priorities and research on the ground.
2. Pilot an accessible monitoring and evaluation (M&E) approach for assessing the influence of research agendas and generating lessons for future application.

Through this analysis, we evaluate both the relationship between the GSRA and research activity in the field and the utility of the methodology, highlighting lessons learnt on how this could be applied moving forwards.

## Methodology

This study employed a desk-based methodology, piloting and implementing a novel mapping approach described below, which used all accepted abstracts from the 2019 and 2022 SVRI Forums. Given the large number of abstracts reviewed, individual abstracts are not described.

This study is grounded in an interpretivist paradigm, which views knowledge as socially constructed and emphasizes the interpretation of meaning within texts [11]. Methodologically, the analysis draws on directed qualitative content analysis, an approach that uses existing frameworks to guide initial coding categories while allowing for inductive insights where data do not fit predefined codes [4]. The 41 GSRA research questions served as an a priori coding framework, and abstracts were mapped to these questions using a structured “best-fit” procedure. Abstracts that did not align with existing GSRA questions were analysed inductively to identify emerging themes, consistent with contemporary qualitative analytic guidance [26].

### The SVRI forums

The SVRI Forum is an abstract-driven conference focused on VAW and VAC and is, to our knowledge, the largest global convening dedicated specifically to this field. The Forum is held biennially, with a delay between 2019 and 2022 due to the coronavirus disease 2019 (COVID-19) pandemic. In 2019, the Forum was held in Cape Town, South Africa, with over 750 attendees . In 2022, it was held in Cancún, Mexico, convening 1021 participants from 99 countries (SVRI 35).

The Forum brings together researchers, practitioners, policy-makers, funders and activists to share emerging evidence and practice-based insights. Participation is geographically diverse, with representation from over 100 countries across recent convenings. The Forum serves as an established platform for knowledge exchange, capacity strengthening and dissemination of new and emerging research on VAW and VAC [12]. The calls for abstracts are issued globally, with options to submit in Spanish, French and English, highlighting the Forum’s commitment to international and multi-lingual participation. Submissions are not restricted to the GSRA priorities, and abstracts are welcome across VAW and VAC research and practice, therefore reflecting a wide range of research foci and contexts.

Access to the abstracts from the 2019 and 2022 SVRI Forums was obtained through collaboration with the SVRI and the publicly available conference materials. While conference data provided opportunities to examine participation by region, methodology and other characteristics, the present analysis focused on thematic alignment with GSRA priorities rather than on geographic distribution or demographic characteristics of presenters.

The selection of the SVRI Forum was also a pragmatic choice: it provided a large, accessible and thematically relevant dataset that could be analysed systematically within the scope and resources of this study. The Forum’s scale, global reach and recognized agenda-setting role in the VAW and VAC sector made it a meaningful and appropriate source for assessing how the GSRA was reflected in current research activity. As the only global conference dedicated exclusively to VAW and VAC research, the SVRI Forum offers a uniquely comprehensive and field-defining view of emerging evidence, methodological trends and research priorities.

### Team

The project was conceptualized and led by E.D. and M.T. In the pilot phase the mapping, coding and analysis was conducted by a doctoral researcher (A.R.R.P.) who was engaged in a secondment fellowship with the SVRI, with a research assistant (S.E.) working with M.T. In the second phase of the project, A.R.R.P. worked with a student (Y.B.Z.) completing an internship with the SVRI. Both Y.B.Z. and A.R.R.P. are fluent in English and Spanish, making it possible to review abstracts in both languages. For abstracts submitted in French, the mapping team used online translation tools with support from the wider SVRI team.

### The mapping process

The mapping process was carried out in two distinct phases: Step 1 – the learning phase – and Step 2 – the research phase. In both steps, abstracts were systematically assessed and matched against the 41 research questions outlined in the GSRA.

The two-step structure was designed to ensure consistency and transparency in how GSRA domains and questions were applied. Step 1 functioned as a methodological learning and calibration phase, allowing the team to test the coding framework, refine decision rules and document how to consistently match the GSRA questions to different abstracts. Step 2 then applied the refined and agreed-upon approach to the full dataset, strengthening comparability across abstracts and improving the reliability of the final classifications. During Step 1, multiple iterations of the mapping approach were tested to identify the most efficient methodology for application.

The GSRA research questions, organized by domain, are presented in Table 1. Domains refer to broad areas of research, while questions represent specific priorities within those domains.

### Step 1: The learning phase

Step 1 involved mapping all abstracts which were *submitted* to the 2019 SVRI Forum (1180 in total). Abstracts were mapped to the domain before mapping to individual research questions. Abstracts were first matched to the most appropriate theme and corresponding research question using a “best-fit” approach. If an abstract did not align with a research question or if it applied to more than one research question, secondary research questions were applied.

Assessing the “best fit” of an abstract evolved during Step 1. We started by identifying the primary aim and thematic focus of the abstract by looking at dominant themes and key words. Once the mapping was completed, the research team evaluated the process and agreed on some key lessons learnt (Table 2). In Step 2 we applied what we had learnt in Step 1, repeating the process with abstracts from the 2022 SVRI Forum.

**Table 2:** Lessons learnt during pilot exercise

Pilot exercise experience	Lesson learnt	Strengthened methodology building on learnings from the pilot
Using submitted abstracts versus accepted abstracts	*Submitted* abstracts are not necessarily representative or relevant to the field, as submitting is open to all.	Only abstracts *accepted* to the SVRI Forum 2022 were used, making the process more efficient and the sample more relevant to the field.
Mapping abstracts to GSRA domains before mapping them to research questions within the domain chosen	Abstracts may be relevant to more than one domain, making this step irrelevant and confusing for later analysis.	Abstracts were matched directly to the GSRA research questions, improving both efficiency and accuracy in the process.
Documenting unmatched abstracts	Some abstracts did not match with a research question.	Using a thematic analysis, categories of unmatched abstracts were created to ensure that emerging themes were noted and the integrity of GSRA questions was maintained.

### Step 2: The research phase

In the second phase, the 348 *accepted* abstracts from the 2022 SVRI Forum were mapped against the 41 GSRA research questions, with questions being categorized as matched (a good fit with GSRA questions), overlapping (fits two GSRA questions) or unmatched (could not be matched to any GSRA questions). During this process, research questions in the GSRA would occasionally be expanded or adapted, but this did not change the substance or intent of the question. Establishing and documenting rules for how domains and questions were applied, and how these evolved over time was a key part of ensuring consistency throughout the process. Colour coding was used to flag abstracts for further review and highlight illustrative examples. An inductive thematic coding process was used to analyse the 113 abstracts not aligned with existing GSRA research questions [32]. The core coding team (A.R.R.P. and Y.B.Z.) met regularly to refine categories and coding frameworks, with E.D. and M.T. providing periodic oversight to maintain consistency and rigour.

We assessed country affiliation for all first authors across the 348 accepted abstracts. The top five countries represented in the submission pool were the USA (98 abstracts), the UK (38), South Africa (35), Mexico (23) and Uganda (12). As an important caveat to this information, it is important to note that the country association for first authors only determines where their institutional association is located or registered and that the countries where research was conducted, interventions were piloted and people were involved were much more diverse. In total, there were 68 countries represented in the country associations. The dominance of institutions based in the USA and UK suggests that institutional leadership in the research presented at the Forum remains concentrated in high-income settings, despite the GSRA’s focus on LMIC priorities.

## Results

This results section is organized around three main themes which emerged during the mapping process: trends in VAW and VAC research presented at the 2019 and 2022 SVRI Forums, highlighting GSRA research priorities that were more or less frequently aligned with abstracts, and emerging topics not yet reflected in the agenda were identified and categorized. By identifying trends, gaps and emerging topics, this paper highlights how the field has evolved over time and where it may be heading.

In this mapping exercise, abstracts were classified as *matched* if they aligned with a single GSRA research question, *overlapping* if they aligned with two questions, and *unmatched* if they did not align with any existing GSRA research question. Unmatched abstracts lacked sufficient detail, fell outside the scope of the GSRA or addressed topics unrelated to the research questions.

### Domain trends between Step 1 and Step 2

**Fig. 1:**
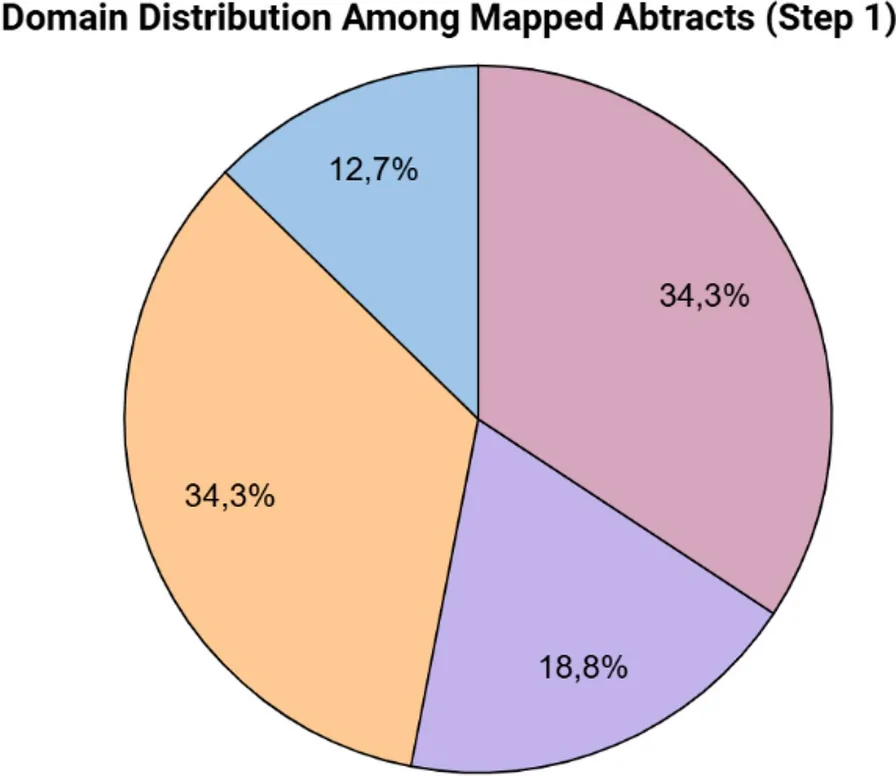
Domain distribution among mapped abstracts in the learning phase

**Fig. 2:**
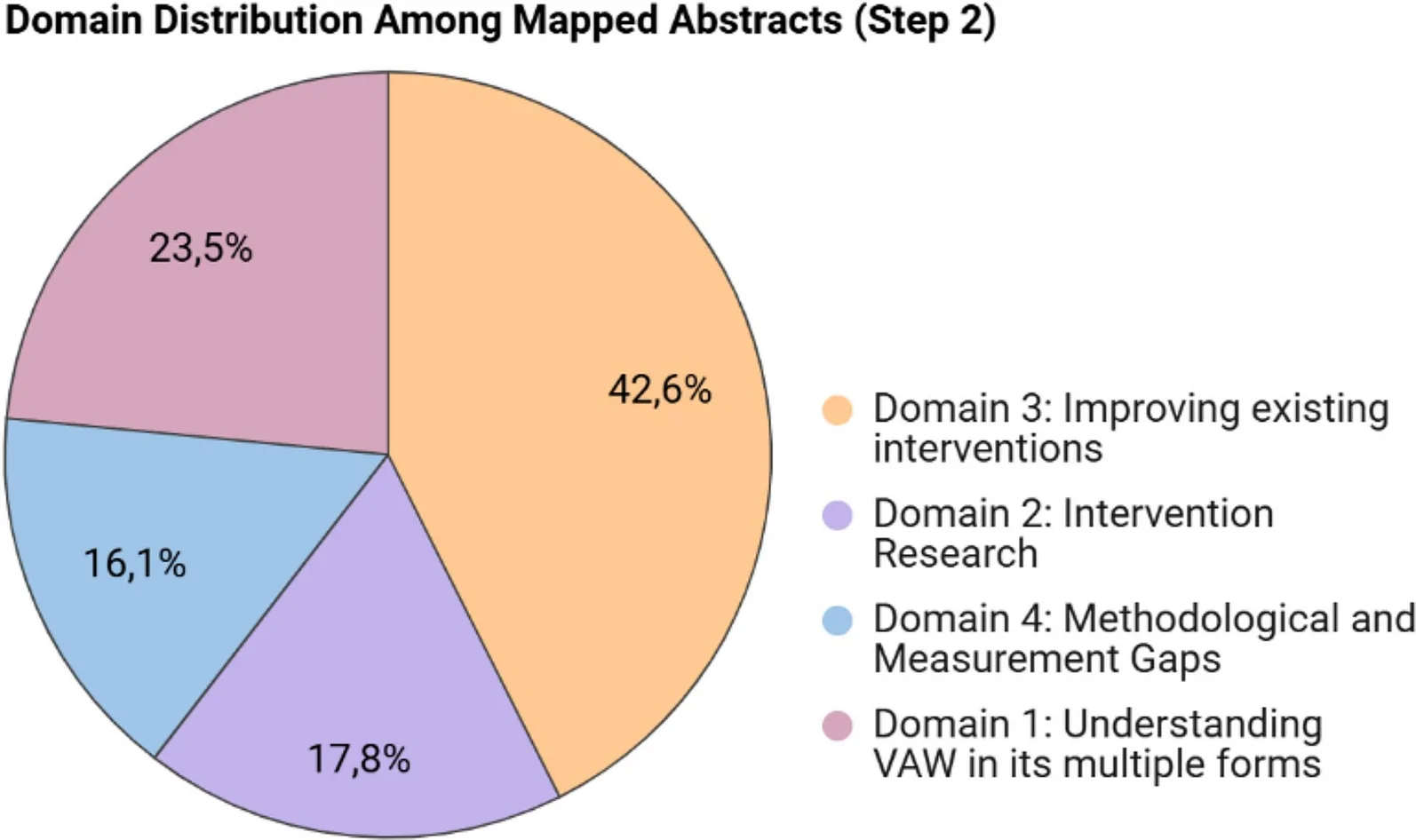
Domain distribution among mapped abstracts in the research phase

Abstracts were most frequently categorized under two domains: Domain 1, understanding violence against women and girls in its multiple forms (34.3% and then 25.1%), and Domain 3, improving existing interventions (34.3% and then 42.8%). The number of abstracts categorized under *Domain 3* grew, from 34.3% to 42.8%, in Step 2. Figures 1 and 2 illustrate the domain distribution among *matched* abstracts in Step 1, the learning phase, and Step 2, the research phase, respectively.

Percentages were calculated by taking the total number of abstracts in the domain and dividing this by the number of total abstracts *matched,* creating a comparable metric, and correcting for abstracts categorized as unmatched in Step 2.

### Research question trends between Step 1 and Step 2

During Step 1, the most popular question was in Domain 1, understanding VAW in its multiple forms. Fifty-nine abstracts, or 15% of abstracts, were matched to a single question (Question 30). This question was also among the most used questions for Step 2, with 5.8% of total abstracts matched being allocated there, showing a downward trend.

During Step 2, the most popular question was in Domain 3, improving existing interventions: In total, 32 abstracts or 14% of abstracts matched were allocated to Question 18. This was also a popular question in Step 1, with 12.4% of all abstracts matched.

### Least used research questions

In both Step 1 and 2, there were several research questions with one or less abstracts matched to it. These included:

- Q36: What types of interventions are effective in preventing IPV and other forms of violence against LGBTQIA+ people?
- Q38: How can police response more adequately address the needs of LGBTQIA+ people reporting IPV non-partner sexual violence and sexual harassment?
- Q15: In IPV prevention interventions inclusive of women and girls with disabilities, should outcome measures be universal, or should some be disability-specific?
- Q14: What research methodologies are most appropriate to measure social norm change in violence prevention interventions?

### Step 2: Distribution of matched and unmatched abstracts

**Fig. 3:**
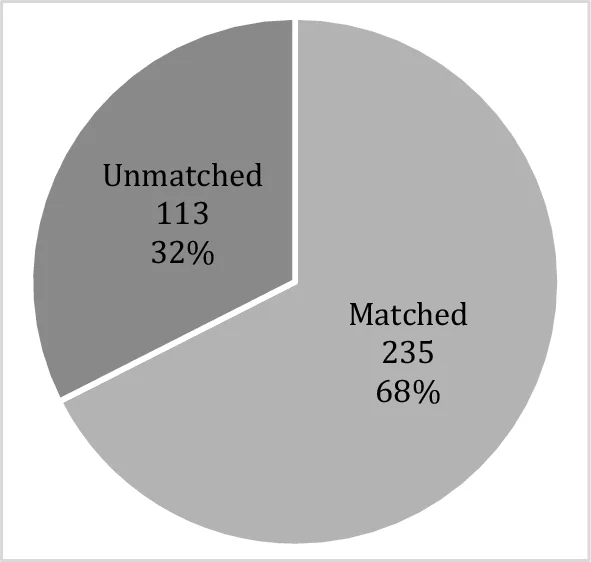
Distribution of matched and unmatched abstracts

In Step 2, 113 abstracts (32.5%) did not match any question in the GSRA, while 235 (67.5%) were matched, as illustrated in Fig. 3.

To illustrate the process, the following three examples demonstrate how abstracts were categorized as matched, overlapping or unmatched.

Example 1: Matched abstract:

- Abstract title: “Technology facilitated GBV: Findings from Asia”.
- Abstract details: Through an analysis of a literature review on technology facilitated gender-based violence (TFGBV) in Asian LMICs, the authors found that TFGBV is shaped by context specific social norms and technological landscapes. The findings support existing evidence that women, girls and sexual minorities (especially those with intersecting marginalized identities) are at higher risk. It also underscores underreporting and disjointed, outdated, or non-existent legal definitions of TFGBV as barriers to enhanced reporting.
- Matching status: Matched with the GSRA question “What is the prevalence of different forms of online and technology-facilitated VAW, and what are the risk and protective factors for experience and perpetration of these types of violence?”
- Notes on matching: The abstract contributed to answering the GSRA research question, by providing localized research.

Example 2: Overlapping abstract:

- Abstract title: “Violence against Refugee LBT Women in Turkey and Lebanon”
- Abstract details: This abstract has a main theme, which relates to the LGBTQIA+ questions in the GSRA. However, it also has a strong focus on sexual violence against refugees in Lebanon and Turkey, which showcase patriarchal, heteronormative social norms. Further, evidence suggests these contexts are increasingly anti-refugee, which can make access to services, support networks and reporting much harder. The research bridges this gap, focusing on a population impacted by conflict and instability.
- Matching status: Overlapping.
- Notes on matching: The question “What are the causes and drivers of violence against LGBTQIA+women?” is insufficient by itself. This is an example of an abstract that requires a secondary question; in this case we can use the question “How do conflict and fragility exacerbate the multiple forms of violence experienced by women and girls?” to better represent this research.

Example 3: Unmatched:

- Abstract title: "Femicide perpetrator’s narratives on gender, violence and identity”.
- Abstract details: The focus of this abstract is to explore narratives perpetrators use to contextualize, justify or legitimize their crimes. The research identifies three key findings: abandonment as a trigger for violence, a self-perception of perpetrators as non-violent and the femicide act as a transference of emotional pain.
- Matching status: Unmatched and categorized in “perpetrator characteristics”.
- Notes on matching: The GSRA lacks questions about perpetrators, as it is rooted in feminist values and is driven by a survivor-centred approach.

### Step 2: Categories of abstracts that did not map on to the GSRA

From a total of 113 unmatched abstracts (out of 348 overall), nine new themes were identified, with a minimum of five abstracts in each. See Table 3 for a full list of emerging categories.

**Table 3:** Description of unmatched categories

New category	Description of new category
Perpetrator characteristics	Focus is directly on perpetrators. Perpetrators are often under-researched due to the feminist nature of VAW/C and VAC work. Includes perpetrator behaviours, experiences and causes.
Reproductive care	Reproductive care or its lack thereof. Includes reproductive violence and coercion.
Response	Response interventions, pathways, services and evaluations. Includes analysis of experiences for survivors, and response pathways and support services.
Cultural understanding and relevant responses	In-depth understanding about specific cultures. This is applied beyond Indigenous groups and is understood as any group with a particular shared culture. Includes explorations of resilience and discrimination against specific groups and localization/adaptation of interventions to specific contexts.
Basic understanding	Prevalence and nature of violence. Includes research about the drivers of violence, including social norms, and longitudinal prevalence research. A forward-looking approach may presuppose basic understanding, but there is still a lot of prevalence research happening.
Conceptual questions	Theoretical conceptual frameworks and their application. Includes philosophical and psychological theories.
Public health and medicine	Research using a public health lens or geared specifically to the medical sector. Includes research on medical procedures or issues, as well as research on accessibility to the health systems.
Researcher and practitioner well-being/education	Research on how to enhance and ensure the well-being and education of researchers and practitioners. Includes trauma-informed practices and training programs to improve the skills of those working in the field.
Politics and funding	Building partnerships, ethical funding and influencing politics. Includes research on influencing and advocacy efforts.

Table 4 presents the distribution of abstracts that did not map onto the GSRA across nine emerging categories, with “basic understanding” as the most prominent, highlighting areas of research not fully captured by the current agenda.

**Table 4:** Distribution of unmatched abstracts in Step 2

Emerging categories	Amount of abstracts
Basic understanding	23
Public health and medicine	15
Response	14
Researcher and practitioner well-being and education	14
Reproductive care	11
Politics and funding	11
Conceptual questions	9
Perpetrator characteristics	8
Cultural understanding and relevant responses	7

## Discussion

The main finding of this study is that, while the GSRA broadly reflects the field of VAW and VAC research, important gaps exist between its stated priorities and the research presented at the SVRI Forums.

There has been a proliferation of research priority-setting exercises in recent years, yet few have attempted monitoring and evaluation to see whether they impact the field or truly reflect what is happening on the ground [3, 25, 30, 47, 51]. A strength of the approach discussed in this paper lies in its ability to expose divergence between current research and the GSRA. For instance, some of the most under-addressed GSRA questions across both Step 1 (the initial learning and calibration phase) and Step 2 (the systematic application of the refined approach) centred traditionally marginalized groups, such as LGBTQIA+ communities and women with disabilities. While the overall number of abstracts concerning these groups remained stable across both rounds, the way they were matched evolved, highlighting where the agenda and research on the ground are not aligned, and where increased attention or support is required.

### Matched abstracts and questions

The questions with most matched abstracts were those with broader framing, and they often served as a catch-all category for abstracts that did not align clearly with more specific GSRA questions. This trend was less pronounced in Step 2, when the refined coding approach was applied more systematically and the introduction of unmatched categories allowed for clearer differentiation. In Step 2, we saw a rise in questions about interventions, adaptation and scale-up. This higher match frequency could reflect an interest in practical and scalable solutions, consistent with the expanding evidence base on what works to prevent VAW and VAC [16, 22, 44].

In general terms, the trends identified around matched abstracts may have been (in part) related to the level of specificity in the questions. This pattern illustrates a broader issue about how specific research priority questions need to be so as to be effective [47]. What types of questions are most useful to those who are using the research agenda and how they are framed are also shaped by broader questions of ownership and positionality. Critiques highlight that research agendas developed without strong LMIC leadership or decolonial perspectives can reproduce imbalances in whose knowledge is valued, reflecting the wider pattern in which Global North institutions often have greater influence over how research priorities are framed [2, 52]. At the same time, reviews of priority-setting processes emphasize that questions need to be sufficiently clear, actionable and aligned with the decision-making needs of those implementing them; otherwise, they risk being too broad to guide investment or too narrow to capture the realities of research practice [25]. Ensuring that questions are both specific and grounded in locally defined knowledge systems is therefore essential for making research agendas usable and relevant in practice.

Our project contributes to this wider discourse by offering a practical way to see how these dynamics play out in real time. By examining how researchers interpret, use and sometimes work around the GSRA questions, we generate insight into where the agenda is functioning well and where questions may need refinement. The emergence of unmatched categories highlights where the field is moving and where future agenda-setting might need to adapt. In this sense, the project provides a form of monitoring that helps identify whether priority questions are specific, actionable and aligned with the evolving concerns of the field.

Questions with lower match frequency focused on LGBTQIA+ populations, disability and social norm measurement across both steps. Rather than indicating a lack of research in these areas, this likely reflects a misalignment between the GSRA’s framing and the existing research landscape. Much of the submitted research focused on intersectional lived experiences of stigma, societal violence or broader exclusion, topics that did not match neatly onto GSRA questions. For example, the disability question focused on disability-specific measurement, and LGBTQIA+ focused on police violence, rather than meaningful inclusion and access. This points to a potential blind spot in the GSRA’s current structure, where some lived experiences are not fully captured despite the participatory nature of its development [10]. Although the GSRA aimed to reflect field-defined priorities, narrowly framed questions may have limited resonance with researchers conducting equity-driven work.

Taken together, these findings underscore the importance of regularly revisiting and refining research priority frameworks to ensure they remain inclusive and responsive to evolving field dynamics. Examples of important considerations that emerge include the specificity of research priority questions and the way traditionally marginalized voices are included and can shape research priorities.

### Unmatched abstracts and emerging categories

The ratio of abstracts matched to the GSRA versus those unmatched is relatively high, which is unsurprising given the aspirational nature of the GSRA. In the unmatched themes, “basic understanding” and “response” were most used, reflecting the GSRA’s forward-looking, prevention focus that potentially excludes prevalence and multi-disciplinary response research, particularly in populations with multiple intersecting marginalized identities. Similarly, research on decolonization was mainly outside the GSRA’s remit. The GSRA question that addresses the impacts of colonization focused specifically on Indigenous men. Most of the accepted abstracts on colonization looked at broader themes, for example, a study on training humanitarian aid workers discussed the prevalence of implicit racialized, post-colonial rhetoric in curricula. This abstract reflected broader trends of research engaging with postcolonial theory. Abstracts such as this one were classified under “cultural understanding and relevant responses”. The increase in the number of abstracts where researchers engage with the VAC/VAW field using the critical lens of postcolonial theory suggests that this focus area is expanding and potentially warrants more attention, including policy and funding support.

Research on practitioners and researchers themselves was also a prominent emerging theme. In our newly defined category “researcher and practitioner well-being/education”, we grouped abstracts which looked at ethics, well-being and educational support, as well as trauma-informed practices, while “politics and funding” included research on partnerships, political influence and feminist funding models. The surge in this area offers insight into how the field is evolving and the shift towards feminist funding models and ethics of care, with potential implications for policy and agenda-setting [24].

Finally, it is important to recognize that the research presented at a conference is largely backward looking (reporting on research already conducted), while priority-setting is forward looking and aspirational. Thus, there is likely to always be some mismatch. It is also important to note that the GSRA is one of several research agendas in the field and, by design, cannot address every research gap or priority. Rather, it serves as a foundational and catalytic effort, intended to have broad reach and inspire collective action on VAW and VAC research in LMICs. In this regard, the observed alignment with GSRA priorities suggests that the agenda is broadly reflected in current VAW and VAC research. The monitoring and evaluation (M&E) research summarized in this paper should be viewed as a springboard for continued inquiry, building on the momentum of the GSRA to further strengthen the evidence base, while acknowledging that ongoing, complementary efforts are essential to fully address the complexity of VAW and VAC.

### Implications for research, policy and agenda-setting

This matching process has the potential to bridge the gap between the current state of the field and the broader aspirations outlined in the research agenda. This approach (or a version of it) offers a practical way to track progress on GSRA research questions and can help organize and summarize existing research for literature reviews. It could also be used to create research briefs that are updated regularly, serving as a living repository of current evidence and highlighting gaps where more research is needed. As this method is accessible and adaptable, there are opportunities to integrate this into the fabric of research events such as conferences. By inviting participants to self-identify which GSRA question their research addresses at the point of submission, the matching process would become more efficient and potentially more accurate, by bringing researchers directly into the process. Conference proceedings could be planned around key thematic areas and increase visibility and dissemination of the research agenda itself, galvanizing a shared commitment to the priorities across the field. Conference organizers and agenda-setting bodies such as the GSRA steering group are well-positioned to coordinate this process, given their access to submissions and their role in shaping research dissemination. This could be operationalized by embedding a simple self-identification step within routine abstract submission systems and periodically compiling and analysing these data as part of standard conference administration. This approach could also help structure feedback from a range of stakeholders on emerging research priorities to be fed into GSRA updates, with community and lived experience of stakeholders informing relevance and emerging needs, and institutional actors supporting the process and integration into formal agenda revisions. This approach could be streamlined through conference integration and may offer a potentially cost–effective method for tracking alignment with GSRA priorities.

The distribution of institutional affiliations also has implications for research equity and agenda-setting. Although research was conducted across 68 countries, institutional leadership remained concentrated in the USA and UK. This pattern reflects broader structural dynamics in global research funding and authorship, where high-income institutions often shape which research questions receive visibility and support [2, 43]. At the same time, the presence of Mexico, Uganda and South Africa among the top five contributing countries at the SVRI Forum reflects two key points. First, it underscores the substantive contribution to the field from select Global South countries, despite persistent structural imbalances. Second, it demonstrates that centring LMIC priorities works. While we can see that progress has been made in terms of who is contributing to knowledge, there is an ongoing need to continue to strengthen leadership, authorship opportunities and institutional capacity within LMIC-based organizations so that research priorities continue to be driven equitably across contexts.

A related consideration is how responsibility for addressing these gaps should be understood within the wider ecosystem of research agendas and the field. Research agendas such as the GSRA are not static directives but part of an ongoing exchange including, but not limited to, those with lived experiences, agenda-setters, researchers, practitioners and funders. Responsibility can be seen as shared between these actors, with each contributing to creating knowledge and building consensus on promising practices in different ways. For example, those involved in developing and stewarding research agendas have a role in ensuring that priorities remain relevant, inclusive and reflective of emerging evidence and lived experiences. Researchers engage with these priorities in ways that make sense for different contexts while also generating insights that can inform future revisions of the agenda. Funders shape which areas gain traction through what they choose to resource. Understanding responsibility in this way reinforces the central point of this paper: alignment between agendas and the field is achieved through a dynamic feedback loop, and monitoring exercises such as this one are essential for making that loop visible and actionable.

The shift in trends shows how quickly the field is evolving and the importance of updating the GSRA periodically to ensure it represents the field and provides a map for where to go next to eliminate VAW and VAC. Unmatched categories identified provide a valuable entry point for future inquiry, particularly for updates of the GSRA. While the GSRA process made strong efforts to include diverse perspectives by monitoring representation and actively centring underrepresented groups during the priority-setting process, this could be further developed [10]. More recent agenda-setting processes such as the African Shared Research Agenda for Ending Gender-Based Violence have pushed the methodology further by incorporating focus groups with individuals who have relevant lived experience [38]. This has strengthened representation and helped refine research priorities in ways that are more inclusive, grounded and reflective of the realities faced by marginalized communities.

### Limitations and opportunities

While this evaluation and influence approach offers valuable insights, it is not without limitations. As with much qualitative thematic analysis, the mapping process involves a degree of subjectivity. To reduce bias, it requires at least two reviewers and multiple rounds of review to ensure consistency as categories are developed and refined. Additionally, the analysis depends on access to abstracts or research that is broadly representative of the field, an important consideration for the generalizability of findings. Another limitation is the nature of abstracts themselves. While they offer concise summaries, they may lack the depth needed to fully capture the nuances of the research. For the purposes of this analysis (focused on identifying broad trends), this level of detail was sufficient.

The current analysis is based solely on abstracts accepted to the SVRI Forums, meaning it does not encompass the full scope of research being conducted on VAW and VAC globally. Expanding the dataset in future exercises could enhance the comprehensiveness and impact of the findings. Where feasible, incorporating abstracts from other VAW and VAC convenings, organizational repositories or publicly available research summaries would broaden the dataset and offer a more representative picture of how current research activity aligns with the GSRA. Expanding the dataset in this way would capture work that may not be submitted to the SVRI Forums but nonetheless contributes to the evidence base informing agenda-setting processes, echoing wider calls for more coordinated and transparent monitoring of VAW and VAC research landscapes [13, 16, 20].

Drawing on the wider network of SVRI partners, including initiatives such as the Safe Futures Hub [31] and other regional or thematic collaborations, could also support access to additional sources of research activity and help build a more systematic approach to data collation. Expanding the dataset in this way would capture research that may not be submitted to the SVRI Forums but nonetheless contributes to global evidence and practice, as highlighted in recent reviews of VAW and VAC research Ullman et al., 2025. A more detailed analysis of abstracts, including methodological approaches, thematic emphases and geographic patterns, would deepen understanding of emerging trends and participation dynamics. This would enhance the depth and utility of future monitoring exercises and strengthen the evidence base for assessing alignment with the GSRA over time.

## Conclusions

This accessible monitoring and evaluation tool demonstrates potential for supporting adaptive learning and sustaining stakeholder engagement within research agendas. With regard to evaluating the impact of the GSRA, the findings demonstrate how the VAW and VAC field is building on key development milestones, promoting inclusive research and bridging gaps between policy and practice. The findings also show where there is divergence between the GSRA and research on the ground, highlighting where GSRA priorities have not yet fully translated into funded research or policy action, pointing to opportunities for improving alignment and uptake.

Future research could centre on exploring these themes and ensuring meaningful policy-level guidance is given to promote research into these areas, through specific calls for action, when updating the GSRA or in future research agendas. The GSRA remains a valuable tool which has had a clear impact on the field – both in terms of highlighting research progress and gaps and being a benchmark for progressive research agenda-setting methodologies.

## Data Availability

The datasets used and/or analysed during the current study are available from the corresponding author on reasonable request.

## Annex 1

### Most popular questions per domain in each round

#### Round 1

**Table:**

Domain 1: Understanding VAW in its multiple forms	Q30: How do different forms of violence cluster in women and girls with greater vulnerability, and what are the characteristics to detect those vulnerable women and girls? Most popular question overall
Domain 2: Intervention research	Q1: What types of interventions can effectively prevent multiple forms of violence, and why?
	Q29: What are the factors underlying successful intervention and prevention programmes aimed at men including Indigenous men and other under-researched populations?
Domain 3: Improving existing interventions	Q18: How can large-scale sector programmes be adapted to optimize their impact on violence prevention and response particularly education health economic development infrastructure and social protection programmes?
	Q20: What are some best practices for ensuring agility and adaptability of VAW interventions, especially those working with marginalized women and girls or operating in complex contexts?
Domain 4: Methodological and measurement gaps	Q23: How do we ensure our research impacts policy and programmes, and how do we measure that impact?

#### Round 2

**Table:**

Domain 1: Understanding VAW in its multiple forms	How do conflict and fragility exacerbate the multiple forms of violence experienced by women and girls?
	How do different forms of violence cluster in women and girls with greater vulnerability, and what are the characteristics to detect those vulnerable women and girls?
Domain 2: Intervention research	Which interventions are most effective at addressing shared risk factors for VAW and VAC in the family environment, leading to a reduction in both types of violence?
Domain 3: Improving existing interventions	How can large-scale sector programmes be adapted to optimize their impact on violence prevention and response, particularly education, health, economic development, infrastructure and social protection programmes? Most popular question
	What are some best practices for ensuring agility and adaptability of VAW interventions, especially those working with marginalized women and girls or operating in complex contexts?
Domain 4: Methodological and measurement gaps	How do we ensure our research impacts policy and programmes, and how do we measure that impact?
	Which analytical approaches (both quantitative and qualitative) are most appropriate for advancing an intersectional approach to research on VAW?

### Least popular questions in each round

#### Round 1

**Table:**

Q24: Do higher costs in resource-intensive violence prevention interventions represent good value for money when taking into account effectiveness in reduction of VAW?	1
Q36: What types of interventions are effective in preventing IPV and other forms of violence against LGBTQIA+ people?	0
Q26: In what ways can innovative technologies and interventions be used to detect and prevent online sexual harassment and online intimate partner violence?	0
Q36: What types of interventions are effective in preventing IPV and other forms of violence against LGBTQIA+ people?	0
Q38: How can police response more adequately address the needs of LGBTQIA+ people reporting IPV, non-partner sexual violence and sexual harassment?	1
Q14: What research methodologies are most appropriate to measure social norm change in violence prevention interventions?	1
Q15: In IPV prevention interventions inclusive of women and girls with disabilities, should outcome measures be universal, or should some be disability-specific?	1

#### Round 2

**Table:**

Q7: What are the cultural, psychological and economic impacts of colonization on Indigenous men and women, and how do these impacts influence their behaviours and experiences in respect to VAW?	0
Q36: What types of interventions are effective in preventing IPV and other forms of violence against LGBTQIA+ people?	1
Q24: Do higher costs in resource-intensive violence prevention interventions represent good value for money when taking into account effectiveness in reduction of VAW?	1
Q38: How can police response more adequately address the needs of LGBTQIA+ people reporting IPV, non-partner sexual violence and sexual harassment?	0
Q15: In IPV prevention interventions inclusive of women and girls with disabilities, should outcome measures be universal, or should some be disability-specific?	0
Q32: What are the best methodologies to measure the long-term impacts of violence prevention interventions, including reduction in VAW and other intended and unintended outcomes?	0
Q10: What are the most effective tools to measure harmful traditional practices against women and girls (including FGM/C, early and forced marriage, crimes committed in the name of honour, dowry-related violence and son preference)?	0
Q21: What methodologies can be used to measure and attribute the impact of multi-component interventions on VAW prevention, reduction or cessation?	1
Q14: What research methodologies are most appropriate to measure social norm change in violence prevention interventions?	1
